# Evaluation of the Reliability of the CCM-300 Chlorophyll Content Meter in Measuring Chlorophyll Content for Various Plant Functional Types

**DOI:** 10.3390/s24154784

**Published:** 2024-07-23

**Authors:** Joelie M. Van Beek, Ting Zheng, Zhihui Wang, Kyle R. Kovach, Philip A. Townsend

**Affiliations:** 1 Department of Forest and Wildlife Ecology, University of Wisconsin-Madison, 1630 Linden Drive, Madison, WI 53706, USA; jmvanbeek2@wisc.edu (J.M.V.B.); zwang896@wisc.edu (Z.W.); kyle.kovach@wisc.edu (K.R.K.); ptownsend@wisc.edu (P.A.T.); 2 Guangdong Provincial Key Laboratory of Remote Sensing and Geographical Information System, Guangdong Open Laboratory of Geospatial Information Technology and Application, Guangzhou Institute of Geography, Guangdong Academy of Sciences, Guangzhou 510070, China

**Keywords:** chlorophyll fluorescence, chlorophyll content meter, plant functional type, CCM-300

## Abstract

Chlorophyll fluorescence is a well-established method to estimate chlorophyll content in leaves. A popular fluorescence-based meter, the Opti-Sciences CCM-300 Chlorophyll Content Meter (CCM-300), utilizes the fluorescence ratio F735/F700 and equations derived from experiments using broadleaf species to provide a direct, rapid estimate of chlorophyll content used for many applications. We sought to quantify the performance of the CCM-300 relative to more intensive methods, both across plant functional types and years of use. We linked CCM-300 measurements of broadleaf, conifer, and graminoid samples in 2018 and 2019 to high-performance liquid chromatography (HPLC) and/or spectrophotometric (Spec) analysis of the same leaves. We observed a significant difference between the CCM-300 and HPLC/Spec, but not between HPLC and Spec. In comparison to HPLC, the CCM-300 performed better for broadleaves (r = 0.55, RMSE = 154.76) than conifers (r = 0.52, RMSE = 171.16) and graminoids (r = 0.32, RMSE = 127.12). We observed a slight deterioration in meter performance between years, potentially due to meter calibration. Our results show that the CCM-300 is reliable to demonstrate coarse variations in chlorophyll but may be limited for cross-plant functional type studies and comparisons across years.

## 1. Introduction

Chlorophyll is an essential compound within the chloroplast, the organelle responsible for housing the photosynthetic process that gives plants their green color. As a vital component of photosynthesis, chlorophyll captures light energy and converts it into chemical energy, which is used to yield glucose and oxygen [1]. Photosynthetic efficiency is impacted by pigment concentrations [2,3,4], thus quantification of chlorophyll content provides insights into plant growth [5] and photosynthetic functioning [6,7], as well as plant response to environmental [8,9] and climatic [10] interactions.

Increasingly, there is a demand for large volumes of rapidly collected chlorophyll measurements, particularly for agronomic applications requiring rapid assessments for nutrient or disease management [11,12]. However, laboratory methods for quantifying pigments, such as high-performance liquid chromatography, may be logistically infeasible for these applications due to both expense and the need for special handling of samples. Chlorophyll can also be estimated from remote sensing imagery [13,14], but calibration and validation of remote sensing maps require large sample sizes, often collected within a short time window to match remote sensing observations [15].

To meet these demands, various types of portable chlorophyll meters have been developed to non-destructively estimate leaf chlorophyll content in situ. Most of these meters infer the chlorophyll content by measuring leaf spectral characteristics, especially in wavelengths between 400 nm and 1000 nm that have distinct pigment absorption, reflectance, or fluorescence features.

Absorptance meters, such as the SPAD-502 chlorophyll meter (Konica Minolta Sensing, Tokyo, Japan) and Opti-Sciences CCM-200plus Chlorophyll Content Meter (Opti-Sciences, Hudson, NH, USA), measure leaf absorptance at light wavelengths near 650 nm and 940 nm (red and near-infrared, respectively). Absorptance meters do not provide a direct output of chlorophyll content, but instead utilize the ratio of transmittance (which yields a ‘chlorophyll content index’ or ‘SPAD unit’, for example) to produce a relative chlorophyll content measurement, which is the sole output of absorptance meters. Regression analyses can be the basis to convert these measurements to units of chlorophyll content [16]. Reflectance meters, like the UniSpec Spectral Analysis System (PP Systems, Amesbury, MA, USA), record leaf reflectance across many wavelengths (ultraviolet, visible, near-infrared). The output of reflectance meter measurements provides multiple data points, allowing for extensive analyses from a single leaf scan.

Chlorophyll meters based on leaf absorptance/reflectance features suffer from a few drawbacks. First, there is a documented decrease in the accuracy of chlorophyll estimations when leaf chlorophyll content increases due to saturation of absorption [17]. Additionally, the indices generated by such meters require careful evaluation through time (e.g., assessment of calibration consistency), and across species and phenology [18]. Finally, meter measurements are affected by factors other than chlorophyll content, such as differences in leaf structure [19] and structural materials of cell walls [20].

Leaf fluorescence measurements have emerged as an alternative to absorptance/reflectance for quantifying chlorophyll content in leaves. Light that is not absorbed by chlorophyll for photochemistry is re-emitted as heat or fluorescence. Fluorescence occurs during de-excitation after a fluorophore is transitioned into a state of excitement due to absorption of light [21,22,23]. Previous studies have shown that the strength of the fluorescence signal closely tracks the leaf chlorophyll content [24,25,26].

Unlike absorptance- and reflectance-based meters that measure light from an external source, fluorescence-based meters measure the light re-emitted from the leaf. Fluorescence meters, also referred to as fluorometers, measure peak wavelengths in the regions of 685–690 nm (red) and 730–740 nm (far-red, near-infrared). Fluorescence ratios of red and far-red regions [23,27,28] enable direct estimates of chlorophyll content based on regression equations. For example, the widely used Opti-Sciences CCM-300 Chlorophyll Content Meter (hereafter referred to as CCM-300) measures emission ratios of red light at 700 nm to far-red emission at 735 nm (chlorophyll fluorescence ratio F735/F700) and estimates chlorophyll content (mg/m^2^) based on equations from Gitelson et al. [26]. To obtain consistent fluorescence measurements, the instrument is calibrated to a purple transparent fluorescent slide with a predetermined value (0.8 for our unit) before each measurement session.

The CCM-300, a portable, non-destructive device, has gained wide usage in field biology due to its ease of use and ability to provide rapid and repeatable measurements. Although the meter has been used to assess plant and ecosystem health in various studies [29,30,31], the accuracy of the instrument has rarely been tested. Additionally, Gitelson’s [26] equations used by the CCM-300 are based exclusively on broadleaf species, suggesting the need for testing on other leaf physiognomic types. Indeed, the Opti-Sciences CCM-300 Chlorophyll Content Meter Operation Manual [32] encourages users to develop unique calibration parameters for vegetation types not represented by the Gitelson [26] equations. However, despite its popularity, the validation of the CCM-300’s reliability across plant functional types is scarce, and we do not know of any published alternative equations for non-broadleaf species. As far as we are aware, our study is the first to evaluate the performance of the Opti-Sciences CCM-300 Chlorophyll Content Meter.

In this study, we assess the reliability of the Opti-Sciences CCM-300. We aim to (1) compare CCM-300 measurements against traditional laboratory chemistry analyses such as spectrophotometry and high-performance liquid chromatography (HPLC), (2) analyze meter consistency between plant functional types, and (3) determine meter reliability across years, assessing whether there is degradation in estimates as the instrument ages.

## 2. Materials and Methods

### 2.1. Materials

**Figure 1:**
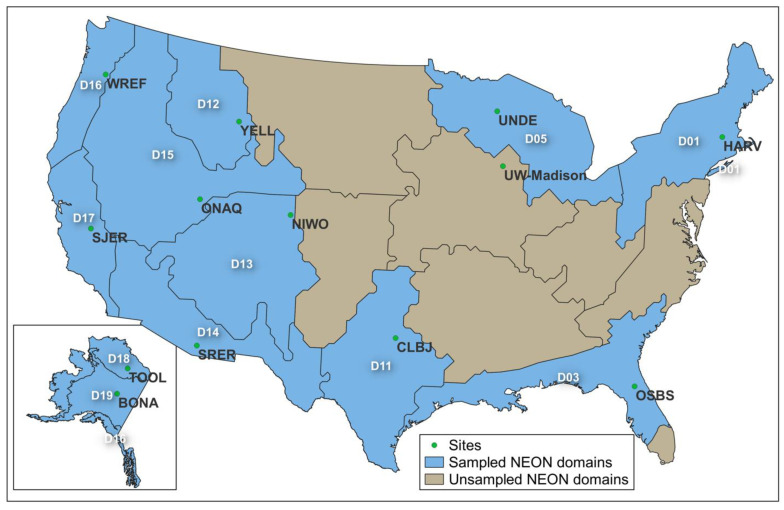
Sample locations. D01–D019 stands for NEON Domain01–Domain019.

In this study, we used two datasets to assess the CCM-300 performance—one contains samples collected on the campus of the University of Wisconsin-Madison (UW-Madison) in 2018 purely for CCM-300 testing purposes, and the other is an opportunistic dataset with samples collected from 12 domains of the National Ecological Observatory Network (NEON) in 2018 and 2019 (Figure 1). Although the NEON data were not gathered with our study in mind, they provided valuable and relevant information, enabling us to maximize the utility of available resources while producing meaningful insights.

The UW-Madison dataset was collected in June 2018, right after the purchase of the CCM-300. We measured the chlorophyll content for 79 foliar samples (22 broadleaves and 57 conifers) using the CCM-300. For each sample, readings were taken from three different places on the leaf, and the average was recorded. After the measurements, the leaves were placed into zip-lock bags with a moist paper towel, sealed, and transported back to the laboratory in a cooler with ice. For conifer needles, we cut the middle section of each needle then scanned the area. For broadleaf species, we used hole punchers to collect 1 cm^2^ leaf material. Following Zhang et al. [33], the sampled leaf material was immersed in a vial with N, N-dimethylformamide (DMF) and stored in a dark refrigerator (4 °C) until leaf chlorophylls were completely extracted. It took around five days to bleach most broadleaf samples. However, for all needles, but especially older ones, more time (~two weeks) was needed for complete extraction. The absorptance of the solvent at 663.8 nm and 646.8 nm was then measured using a Genesys 5 spectrophotometer (Thermo Electron Corp.: Waltham, MA, USA) and the chlorophyll content was estimated using the equations derived by Wellburn [34]. These measurements were referred to as laboratory spectrophotometry.

(1) $$C_{a}=12A_{663.8}-3.11A_{646.8},$$

(2) $$C_{b}=20.78A_{646.8}-2.43A_{663.8},$$

(3) $$C\;=10\left(C_{a}+{\;C}_{b}\right)\times v/s,$$

where C_a_ is the content of chlorophyll a in µg/mL^−1^, C_b_ is the content of chlorophyll b in µg/mL^−1^, v is the volume of the solvent (5 mL in this study), and s is the area of the leaf sample in cm^2^. C is the content of total chlorophyll in mg/m^2^.

To further validate the laboratory spectrophotometry measurements, we flash-froze leaf material from 44 samples (18 broadleaves and 26 conifers) out of the 79 samples in liquid nitrogen and transported them in a −20 °C freezer to the University of Minnesota-Twin Cities, where the chlorophyll content was measured using high-performance liquid chromatography (HPLC) (Agilent 1200 Series HPLC; Agilent Technologies, Santa Clara, CA, USA). HPLC methods are described by Schweiger et al. [35].

Samples from (NEON) were collected for a large-scale study that utilizes imaging spectroscopy to map foliar functional traits [36]. As part of the functional trait measurements, the same CCM-300 instrument was used to rapidly obtain leaf chlorophyll content in the field. Following the NEON sampling protocol [36], measurements were averaged over five leaves in the field. Among these, two to three leaves were immediately flash-frozen to −80 °C in liquid nitrogen. The flash-frozen samples were then kept in a −20 °C freezer and transported to the University of Minnesota-Twin Cities for HPLC measurements. Samples measured using HPLC were referred to as ‘HPLC’.

This NEON dataset includes 244 samples (104 broadleaves, 129 conifers, 11 graminoids) and provides a valuable opportunity to evaluate the performance of the CCM-300 for different plant functional types across two years.

### 2.2. Methods

In each of our analyses, we used methods of laboratory chemistry (HPLC and/or laboratory spectrophotometry) as our “gold standard” due to the reliable, consistent results that these methods have yielded over time [34,37].

We first utilized a paired, two-tailed T-test to compare CCM-300 measurements to HPLC and/or laboratory spectrophotometry results, as well as measurements between HPLC and spectrophotometry. We then used linear regression to further explore the relationships between CCM-300 and HPLC measurements for different plant functional types and across years. Statistical analyses were performed using R (Stats library, R version 4.3.1).

Before all analyses, we removed outliers outside of the interval defined by Equation (4) [38] for each measurement type (CCM-300, HPLC, and laboratory spectrophotometry).

(Q1 − 1.5IQR, Q3 + 1.5IQR), (4)

where Q1 is the first quartile found by calculating the median of the lower half of data presented in numerical order. Q3 is the third quartile found by calculating the median of the upper half of data presented in numerical order. IQR is the interquartile range, calculated by taking the difference between Q3 and Q1. Sample data after outlier removal are grouped by measurement type pairs in Table 1.

#### 2.2.1. Paired, Two-Tailed T-Test

We first conducted the T-test for the 41 samples that were common across all three measurement types. We then performed the T-test for all the paired samples in the [CCM-300, HPLC] and [CCM-300, Spec] pairs. We calculated the mean absolute difference between measurement types for each pair following Equation (5).

(5) $$\frac{\sum \left|X-Y\right|}{n},$$

where X and Y correspond to values of different measurements for a same sample, and n is the total number of samples per pair.

The significance level (*p*-value) from the T-test is affected by the sample size. Due to dramatic differences in sample sizes for paired data (Table 1, 165 + 96 vs. 72 vs. 41), it is difficult to compare their T-test results (significance levels). To obtain more comparable results, we iteratively subsampled 41 samples randomly from [CCM-300, HPLC] and [CCM-300, Spec] pairs to match the sample size of [HPLC, Spec] pairs. We ran 1000 permutations of T-tests for each pair and calculated the *p*-values for each permutation.

#### 2.2.2. Linear Regression Analysis

We created simple linear regression models to compare HPLC and CCM-300 measurements for different plant functional types with the CCM-300 measurements as the independent variable (x) and HPLC results as the response (y). We also performed the linear regression for broadleaf and conifer samples for 2018 and 2019 separately. We calculated the following evaluation metrics to quantify the quality of our regression models: 

Correlation coefficient (*r*):

(6) $$\frac{\sum (x_{i}-\bar{x})\left(y_{i}-\bar{y}\right)}{\sqrt{\sum {\left(x_{i}-\bar{x}\right)}^{2}\sum {\left(y_{i}-\bar{y}\right)}^{2}}},$$

Root mean square error (RMSE):

(7) $$\frac{\sqrt{\sum {\left(y_{i}-y_{p}\right)}^{2}}}{n},$$

where y_i_ is observed values, y_p_ is predicted values, and n is the number of observations.

Bias:

(8) $$\frac{\sum (\hat{\theta }-\theta )}{n},$$

where $\hat{\theta }$ is predicted values, θ is observed values, and n is the number of observations.

Our tests are a comparison of CCM-300 measurements to laboratory chemistry values. Because our data were gathered opportunistically, each analysis that we conducted does not include either HPLC or Spec data.

## 3. Results

### 3.1. CCM-300 Measurements vs. Laboratory Chemistry Measurements

Statistics for the paired samples are summarized in Table 2.

#### 3.1.1. Paired T-Test for 41 Samples with All Three Measurements

**Figure 2:**
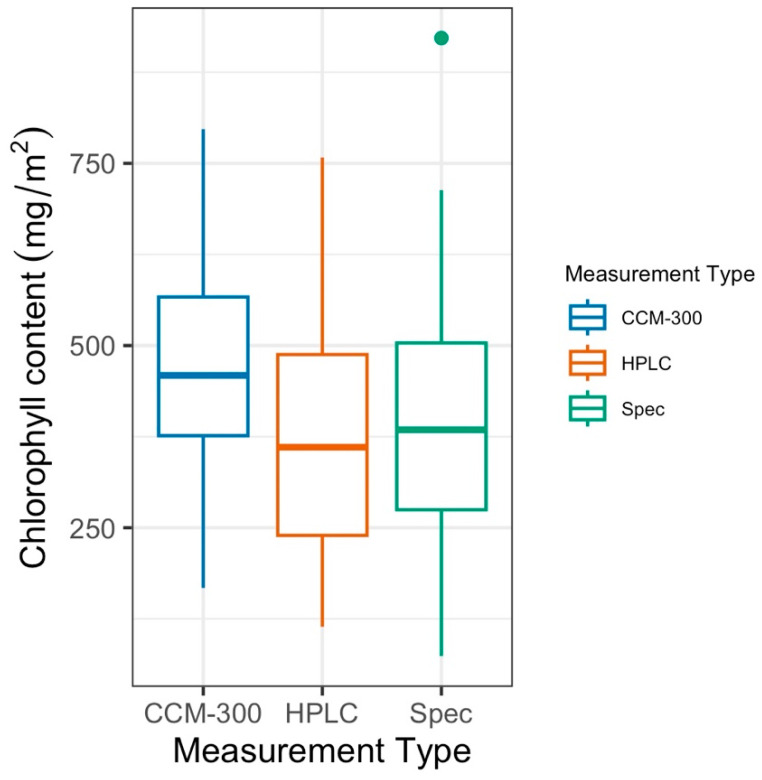
Chlorophyll concentrations (mg/m^2^) by measurement type for paired and presented 41 samples common to all measurement types.

When comparing samples that were subjected to each of the three measurement types (*n* = 41), the data showed a higher median for CCM-300 values than both HPLC and laboratory spectrophotometry (Spec) (Figure 2). The HPLC median was lower than the laboratory spectrophotometry median.

**Figure 3:**
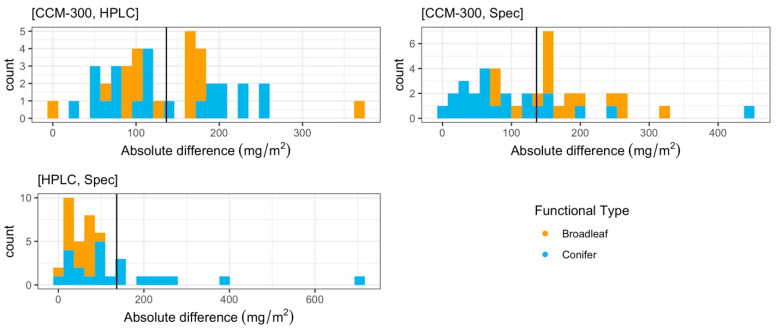
Distribution of absolute differences (mg/m^2^) between specified measurement types for the common 41 samples. Mean absolute difference values are represented by the solid black line.

The results of a paired T-test restricted to these pairs showed that the difference between CCM-300 and HPLC was significant (*p* = 2.20 × 10^−5^). There was also a significant difference between CCM-300 and laboratory spectrophotometry (*p* = 0.005). We did not see a significant difference between HPLC and laboratory spectrophotometry (*p* = 0.32). The mean absolute difference for [CCM-300, HPLC], [CCM-300, Spec], and [HPLC, Spec] was 136.44 mg/m^2^, 131.49 mg/m^2^, and 101.99 mg/m^2^, respectively (Figure 3). [HPLC, Spec] in particular is skewed to the right due to a few samples that have a very high absolute difference, spiking the average for this pair. 

#### 3.1.2. Paired T-Test of All Paired Samples

**Figure 4:**
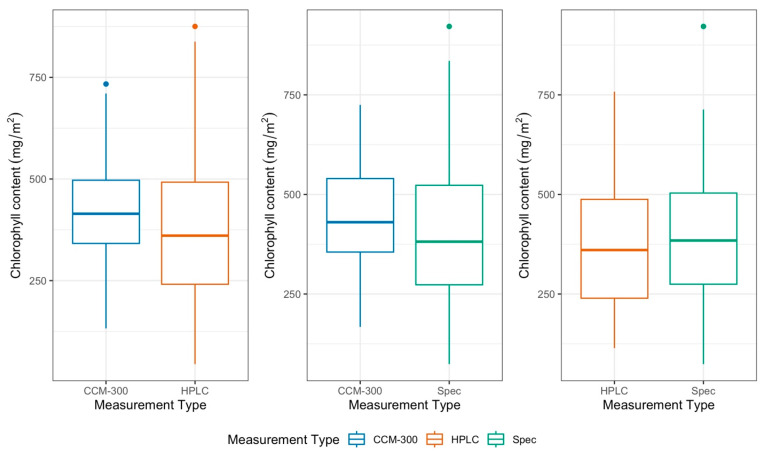
Chlorophyll concentration (mg/m^2^) by measurement type of all paired data before permutations.

**Figure 5:**
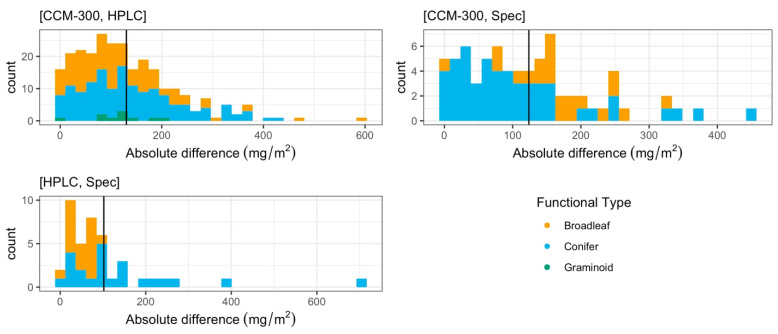
Distribution of the absolute differences (mg/m^2^) between different measurement types for all paired data. Mean absolute differences are represented by the solid black line.

More paired measurements were available for analysis than the 41 common observations represented in Figure 2. The patterns of distribution consisting of all available paired data (Figure 4) were consistent with the distribution of common data points in Figure 2. The results of the paired T-test for all available pairs showed that there was a significant difference between CCM-300 and HPLC (*p* = 2.05 × 10^−5^). There was also a significant difference between CCM-300 and laboratory spectrophotometry (Spec) (*p* = 0.047). There was no significant difference between HPLC and laboratory spectrophotometry (*p* = 0.32). The mean absolute difference for [CCM-300, HPLC], [CCM-300, Spec], and [HPLC, Spec] was 130.26 mg/m^2^, 123.73 mg/m^2^, and 101.99 mg/m^2^, respectively (Figure 5). Each of the pairs was skewed to the right to some extent with [CCM-300, HPLC] and [HPLC, Spec] being impacted by particularly high absolute differences.

#### 3.1.3. Iterative Subsampling for Randomized, Permutated Analysis

**Figure 6:**
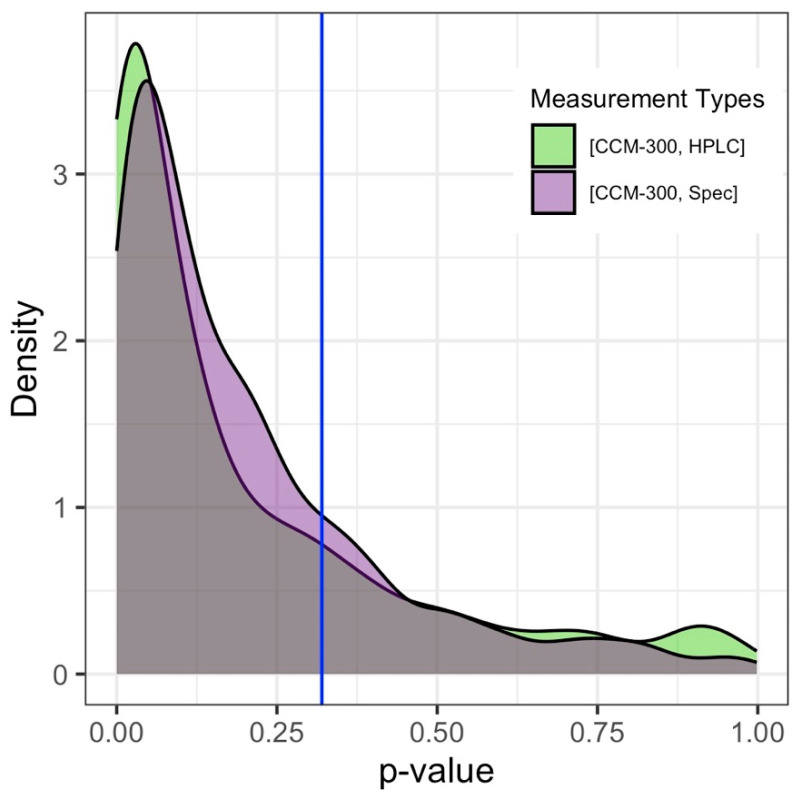
Density plot of *p*-values for (*n* = 41) common paired measurement type samples after 1000 subsampling permutations. The *p*-value for [HPLC, Spec], which has only 41 observations and is therefore not subsampled, is represented by the solid blue line at 0.32.

The distribution of *p*-values from 1000 permutations is shown in Figure 6. We observed similar distribution patterns for *p*-values from [CCM-300, HPLC] and [CCM-300, Spec]. The *p*-value that occurred for [CCM-300, HPLC] most frequently after 1000 permutations was 0.03. The *p*-value that resulted most often for [CCM-300, Spec] after 1000 permutations was 0.047. In comparison, the *p*-value for the [HPLC, Spec] pair was 0.32.

### 3.2. CCM-300 Measurements for Different Plant Functional Types

#### 3.2.1. CCM-300 Measurements for Different Plant Functional Types

We further classified our data into three groups—broadleaf, conifer, and graminoid based on a structural–functional approach, as described by Box [39]. Our original data included a shrub functional type which we reclassified into broadleaf or conifer based on the species. The data available for this part of our study did not include results from laboratory spectrophotometry.

**Figure 7:**
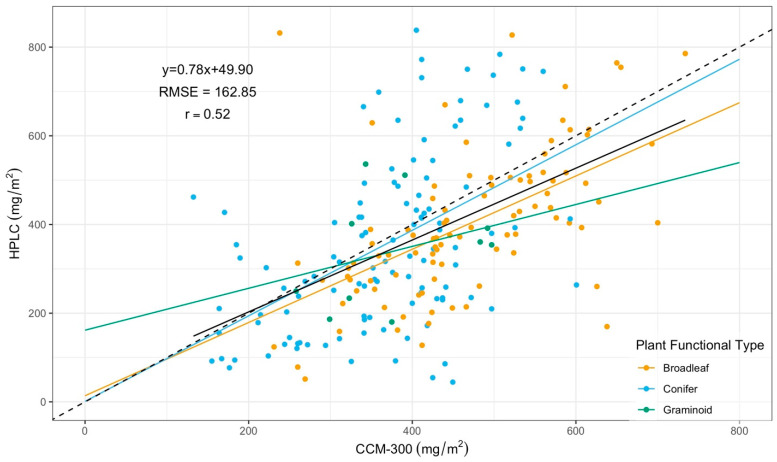
Scatter plot with CCM-300 measurements as X and HPLC as Y colored by different plant functional types. The dashed line is the 1:1 line. The solid black line is the regression trendline with data from all plant functional types. Colored trendlines correspond to plant functional types in the figure legend. The equation, r, and RMSE values in the plot are for the pooled regression model.

Mean and standard deviation values for [CCM-300, HPLC] pairs separated by plant functional type are presented in Table 3. The CCM-300 mean was higher than HPLC for every plant functional type. The linear regression results between CCM-300 and HPLC for all functional types are shown in Figure 7. Overall, our data gathered closely around the 1:1 line. There was a strong correlation between CCM-300 and HPLC measurements for both broadleaves (r = 0.55) and conifers (r = 0.52), but the correlation was weak for graminoids (r = 0.32) (Table 4). Interestingly, the model for graminoids yielded the smallest RMSE (127.12) across all plant functional types, indicating its ability to capture the proper magnitude of chlorophyll content.

Positive bias between CCM-300 measurements and HPLC for all plant functional types (78.41 mg/m^2^, 13.99 mg/m^2^, and 38.11 mg/m^2^ for broadleaf, conifer, and graminoid, respectively) indicated that CCM-300 generally overestimated chlorophyll content for the tested samples. The overestimation was most severe for broadleaf, followed by graminoids, and the least for conifers. Additionally, we observed meter saturation above 625 mg/m^2^ for conifers.

#### 3.2.2. Needle Age Analysis

**Figure 8:**
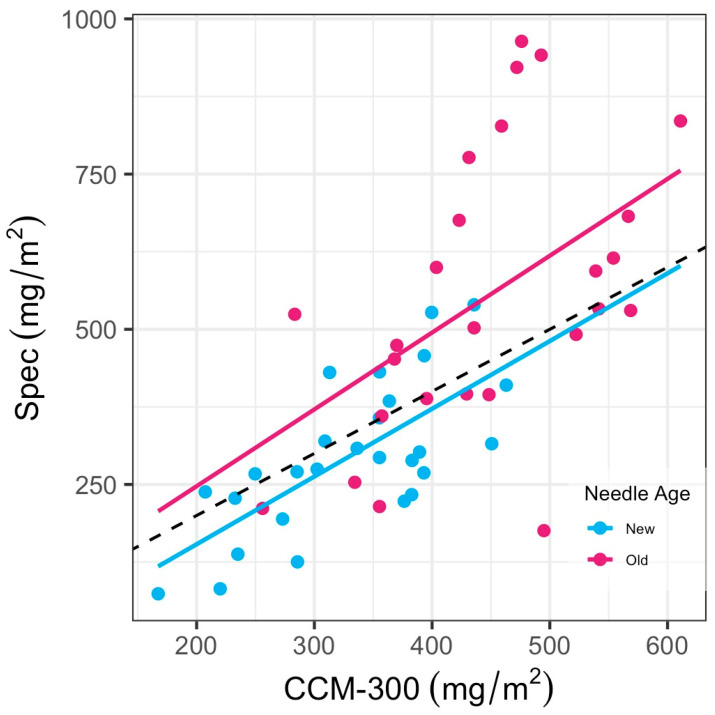
Comparison of new and old needles for CCM-300 (mg/m^2^) vs. Spec (mg/m^2^).

The UW-Madison dataset recorded needle age during the sample collection and enabled us to explore how needle age affects the CCM-300 measurements. We used 51 conifer samples with both CCM-300 and Spec measurements to further study the effects of age. It can be seen in Figure 8 that the CCM-300 meter performed well for new needles but appeared inconsistent for old needles. There was a strong correlation between CCM-300 and laboratory spectrophotometry (Spec) for new needles (Table 5). A similar trend was observed for the HPLC measurements from the UW-Madison dataset (*n* = 26, Figure S1).

Positive bias for new needles (34.46 mg/m^2^) indicated that the CCM-300 mostly overestimated chlorophyll content in the analyzed samples. Negative bias for old needles (−105.62), however, indicated that the CCM-300 underestimated chlorophyll content in these samples.

### 3.3. CCM-300 Performance across Years

**Figure 9:**
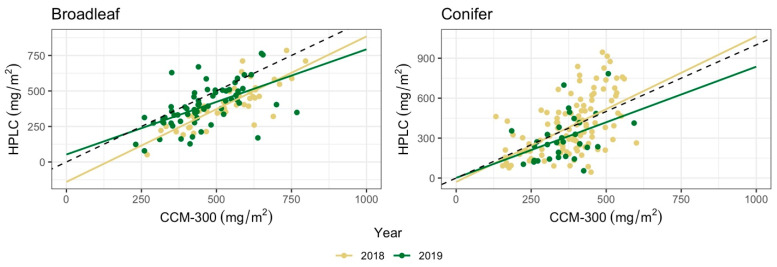
Scatter plots of years for [CCM-300, HPLC] samples compared by functional type. The black dashed lines indicate a 1:1 trendline.

There was a positive linear relationship for both years and both functional types in [CCM-300, HPLC] sample pairs (Figure 9). We observed strong correlations between CCM-300 and HPLC measurements for broadleaf species in both years, and conifers in 2018 (r > 0.5) while the correlation was weaker for conifers in 2019 (Table 6). For conifer samples, the CCM-300 appeared to saturate around 625 mg/m^2^ for all measurements while HPLC measurements can reach values above 900 mg/m^2^ (Figure 9). The CCM-300 performed better for broadleaf species with higher *r* and lower RMSE compared to conifers in both years.

Positive bias for broadleaf samples analyzed in 2018 and 2019 (127.27 mg/m^2^ and 66 mg/m^2^, respectively) indicated that the CCM-300 typically overestimated chlorophyll content in both years, but more so in 2018. For conifer samples, negative bias in 2018 (−6.28 mg/m^2^) and positive bias in 2019 (57.63 mg/m^2^) indicated that the CCM-300 mildly underestimated chlorophyll content in 2018, but generally overestimated in 2019. The CCM-300 performance was inconsistent between years.

## 4. Discussion

### 4.1. CCM-300 Measurements vs. Laboratory Chemistry Measurements

Based on the results of each of our paired T-tests, we found no statistically significant difference between HPLC and laboratory spectrophotometric (Spec) estimates of chlorophyll content (*p* > 0.1). Both laboratory chemistry methods are reliable for obtaining chlorophyll content (Figure 2 and Figure 4). The biggest discrepancies between HPLC and Spec measurements are from old conifer samples (Figure 3, pair [HPLC, Spec]). Old needles usually have higher chlorophyll content [40] and it takes a longer time to fully extract the chlorophyll from the needle samples. During HPLC analyses, all samples were subjected to the same extraction procedure. While the extraction time is enough for most samples, it may not be for a few old needles with exceptionally high chlorophyll contents, which may cause discrepancies between HPLC and Spec.

In contrast, we observed a significant difference between both CCM-300 and HPLC, and CCM-300 and laboratory spectrophotometry (Spec). Comparison of iteratively subsampled paired data (with 41 samples), all pairs, and peak values of permutation *p*-value distribution did not influence the significance of paired T-test results, further supporting our inferences regarding the continued reliability of laboratory chemistry analyses and the potential limitations of the CCM-300. Our results are significantly different at α = 0.05, but the significance would change at α = 0.01 for some pairs. This change in alpha would result in no statistically significant difference for [CCM-300, Spec] pairs from all available paired data, as well as [CCM-300, HPLC] and [CCM-300, Spec] pairs from the permutation analysis (Figure 6).

Additionally, for NEON data, CCM-300 measurements represented the average conditions of five samples while the laboratory chemistry only tested two to three samples. This mismatch could contribute to the observed difference between CCM-300 measurements and laboratory chemistry results.

Despite the differences between CCM-300 and HPLC in the paired T-test, the regression model performed reasonably well when predicting HPLC using CCM-300 measurements (Figure 7). The correlation coefficient (r) for [CCM-300, HPLC] was 0.52. Although the CCM-300 did not match HPLC exactly, it is able to track the variation in chlorophyll content. Nevertheless, laboratory chemistry analyses could produce more reliable estimates.

### 4.2. CCM-300 Measurements for Different Plant Functional Types

The grouping of data around the 1:1 line in Figure 7 implies that the CCM-300 produces reasonable results for chlorophyll content across the plant functional types analyzed in this study. A regression relationship can be used to “correct” the trend in the CCM-300 data, but no single regression is suitable across plant functional types (Table 4). This observation has been seen in other meters and has raised concern in the literature [17,41]. Richardson et al. [17] encourage users of absorptance-based portable chlorophyll meters to consider the need for species-specific calibration when leaf structure varies among samples, while Gamon and Surfus [41] concluded that the chlorophyll index used when analyzing leaf samples with a reflectance-based meter varies between species, specifically those with dissimilar leaf structures.

The meter performed the best for broadleaf species, followed by conifers, but poorly for graminoids. The poor performance for graminoids is likely due to the small variation in range (356 mg/m^2^ for graminoids, compared to 780.35 mg/m^2^ for broadleaf and 830.85 mg/m^2^ for conifer) represented by 10 samples for this functional type in our dataset. 

We observed meter saturation around 625 mg/m^2^ (Figure 7) for conifers, indicating a deterioration in meter sensitivity and accuracy in measuring needles with chlorophyll content beyond this threshold. This saturation is the worst for old needles (Figure 8) where our regression model has the highest RMSE (222.48 mg/m^2^) (Table 5). We suspect that differences in leaf anatomy, likely in the cuticle and epidermis, resulted in the saturation we observed. Needle leaves are known to have thicker and denser external layers compared to broadleaves; thus, the tough structure of old needles likely contributes to the saturation. Lhotáková et al. [42] evaluated the effects of leaf structure on meter outputs and determined that the assessment of needle leaves has several constraints, and it is necessary to perform other biochemical assessments, such as spectrophotometry, for determination of chlorophyll content in needle leaves.

While meter measurements suffered from saturation for conifer samples, measurements are mostly reliable for broadleaves. This is not surprising, since the CCM-300 is based on the work of Gitelson et al. [26], which used broadleaf species exclusively. To the best of our knowledge, Opti-Sciences has not updated the equations utilized by the meter. Our recommendation is to primarily use the CCM-300 for broadleaf species. When analyzing coniferous species with the meter, it is advisable to supplement the measurements with laboratory chemistry to account for any discrepancies [42].

Other studies have reported potential factors contributing to the varied performance of absorptance-based portable chlorophyll meters. Richardson et al. [17] suggest that the size of the measurement area on a given device has an impact on the output of hand-held chlorophyll meters. However, fluorescence-based meters, such as the CCM-300, do not suffer from this drawback, as fluorescence is an active technique measuring light actively emitted from the leaf itself. This does not require the entirety of the measurement window to be filled by a sample, allowing for the measurement of very small leaves. Various studies report that absorptance-based meters also produce less accurate estimations as chlorophyll content increases [17,19,43,44], which is consistent with the results of our study using the CCM-300. It is not surprising for the CCM-300 to be impacted by meter saturation, as other types of handheld devices experience the same phenomenon.

### 4.3. CCM-300 Performance across Years

Based on the results of our analysis between years, the correlation coefficients (*r*) suggest that the CCM-300 captured chlorophyll variation better in 2018 than in 2019 (Table 6). This could be attributed to the lack of hardware calibration for the meter, and/or a deterioration in the quality of the calibration slide used before sample collection. After extensive usage in tough field conditions, the quality of the calibration slide may deteriorate. This could potentially cause drifts in the calibration and may have led to a worse performance of the meter in 2019.

As well, it is plausible that a larger overall sample size with greater uniformity among the sample size of all groups would yield results that are more consistent between both years and functional types. For broadleaf samples, the sample size from 2018 (*n* = 47) was slightly less than 2019 (*n* = 65). Within conifer samples, 2018 (*n* = 111) has significantly more samples than 2019 (*n* = 31).

### 4.4. Study Limitations

The Opti-Sciences CCM-300 provides reasonable results for chlorophyll content if users desire measurements of coarse-scale relative variations. However, broad applications of the CCM-300 for precise estimates may be problematic. Our study used opportunistic data and we cannot rule out that a larger sample size would better validate the precision of CCM-300 measurements, thus our findings may not fully capture the variability in CCM-300 performance. We also provide no absolute measure of accuracy for the HPLC and laboratory spectrophotometric analyses. Regardless, this study provides important evidence that users must carefully evaluate data from the CCM-300 for their applications.

Due to the expense of laboratory chemistry, our study was limited, to an extent, by cost. It is not realistic for many researchers to complete laboratory analyses for every sample in a study, thus portable chlorophyll content meters can be used in certain scenarios as a cost-effective, non-destructive method.

## 5. Conclusions

Our study utilizing opportunistic data suggests that the Opti-Sciences CCM-300 can produce reasonable results for estimating chlorophyll content, but is limited, especially if comparing measurements across functional physiognomic types. Laboratory chemistry analyses continue to be the most reliable method for measurement of chlorophyll content. The CCM-300 is most compatible with broadleaf species and is likely least reliable in measurements of old needles due to meter saturation. There is mild deterioration of the CCM-300 performance between years which could be due to calibration drift.

Moving forward, users of the CCM-300 for broad scale studies, such as in situ measurements to support remote sensing, should conduct a dedicated study with a large sample size composed of many samples from a wider variety of functional types. When measuring needle leaves, we recommend using equations, such as those represented in Table 5, to correct for the large bias in the CCM-300 measurements. It is best practice, however, for users to develop their own equations tailored specifically to their studies.

It may be helpful for those planning future chlorophyll meter studies to consider that chlorophyll is not uniformly distributed within a leaf, impacting meter outputs depending on where the measurement is taken [45]. It is recommended that multiple measurements be taken from each leaf then averaged to improve the accuracy of chlorophyll estimation [32,43]. When doing so, it is best practice to complete measurements of the same leaf within 3 min of the initial measurement to minimize the effects of chloroplast migration on meter outputs [32]. The CCM-300 Chlorophyll Content Meter Operation Manual [32] also recommends that ambient temperatures be considered when taking sample measurements, as this factor may have the potential to influence measurement results.

It is important to adjust measurement parameters to suit the study at hand, but we recommend completing studies with multiple replications of each sample. The CCM-300 includes a range of measuring options that allows users to average between 2 and 30 measurements. Samples with a lower fluorescence emission signal strength may require more measurements [32].

Finally, one should take note of the conditions of the slide used to calibrate the CCM-300 meter. After repeated use, the calibration slide may undergo wear and tear that includes scratches, dents, and loss of adherence to the fluorescence coefficient it was once measured to be. Integrating an internal calibration slide into the meter would ensure consistent slide quality, and including a slide replacement schedule would enhance usability.

We hope that, ultimately, this study provides guidance on CCM-300 applications for large-scale chlorophyll content quantification in support of calibration and validation of a forthcoming generation of spaceborne imaging spectrometers, such as Surface Biology and Geology (SBG) and Copernicus Hyperspectral Imaging Mission for the Environment (CHIME) that will be used to map at high resolutions (30 m) seasonal chlorophyll worldwide at monthly or better time scales.

## Figures and Tables

**Table 1:** Sample sizes for measurement type, year, and plant functional type presented in terms of paired sample data.

	2018			2019
	[CCM-300, HPLC]	[CCM-300, Spec]	[HPLC, Spec]	[CCM-300, HPLC]
Broadleaf	46	21	18	65
Conifer	109	51	23	31
Graminoid	10	0	0	0
Total	165	72	41	96

**Table 2:** Sample size, mean (mg/m^2^), standard deviation (mg/m^2^), and median (mg/m^2^) for pairs of [CCM-300, HPLC], [CCM-300, Spec], and [HPLC, Spec] measurements.

	[CCM-300, HPLC]	[CCM-300, Spec]	[HPLC, Spec]
n	261	72	41
$\underline{x}$	420.0, 377.69	439.68, 403.1	375.57, 400.72
s_x_	119.59, 181.23	132.07, 175.2	152.92, 161.71
median	414.5, 360.67	430.33, 381.48	360.46, 384.39

**Table 3:** Mean (mg/m^2^) and standard deviation (S.D.) (mg/m^2^) values for CCM-300 and HPLC by plant functional type.

		Mean (mg/m^2^)	S.D.
Broadleaf			
	CCM-300	481.19	115.06
	HPLC	402.78	156.85
Conifer			
	CCM-300	374.44	102.93
	HPLC	360.45	200.09
Graminoid			
	CCM-300	378.70	85.60
	HPLC	340.59	126.25

**Table 4:** Regression data for CCM-300 vs. HPLC by functional type.

Functional Type	Regression	r	RMSE (mg/m^2^)
Broadleaf	Y = 0.75x + 41.53	0.55	154.76
Conifer	Y = 1.01x − 16.29	0.52	171.16
Graminoid	Y = 0.47x + 161.65	0.32	127.12

**Table 5:** Regression data for CCM-300 (mg/m^2^) vs. Spec (mg/m^2^) by needle age.

Needle Age	Regression	r	RMSE (mg/m^2^)
New	Y = 1.09x − 64.61	0.70	91.50
Old	Y = 1.24x − 0.32	0.49	222.48

**Table 6:** Regression data for CCM-300 vs. HPLC by functional type and year, where n represents the number of samples.

	n	Regression	r	RMSE (mg/m^2^)
Broadleaf				
2018	47	Y = 1.02x − 140.38	0.81	155.88
2019	65	Y = 0.74x + 52.65	0.56	140.35
Conifer				
2018	111	Y = 1.09x − 29.06	0.54	181.18
2019	31	Y = 0.83x + 1.57	0.42	164.27

## Data Availability

Detailed sample information and chlorophyll content measurements are all available online at Zenodo: https://zenodo.org/doi/10.5281/zenodo.10581895 (accessed on 29 January 2024).

## Supplementary Materials

The following supporting information can be downloaded at: https://www.mdpi.com/article/10.3390/s24154784/s1, Figure S1: HPLC measurements from the UW-Madison dataset (*n* = 26).
