# Supplementary material for: Evaluation of the Reliability of the CCM-300 Chlorophyll Content Meter in Measuring Chlorophyll Content for Various Plant Functional Types
Source: Sensors (Basel). 2024 Jul 23;24(15):4784. doi: 10.3390/s24154784 (PMC11314797; doi:10.3390/s24154784)
Supplement: Supplementary file 1 [file sensors-24-04784-s001.zip › sensors-3088912-supplementary.pdf]

sensors

Supplementary Materials

Evaluation of the Reliability of the CCM-300 Chlorophyll Content Meter in Measuring Chlorophyll Content for Various Plant Functional Types

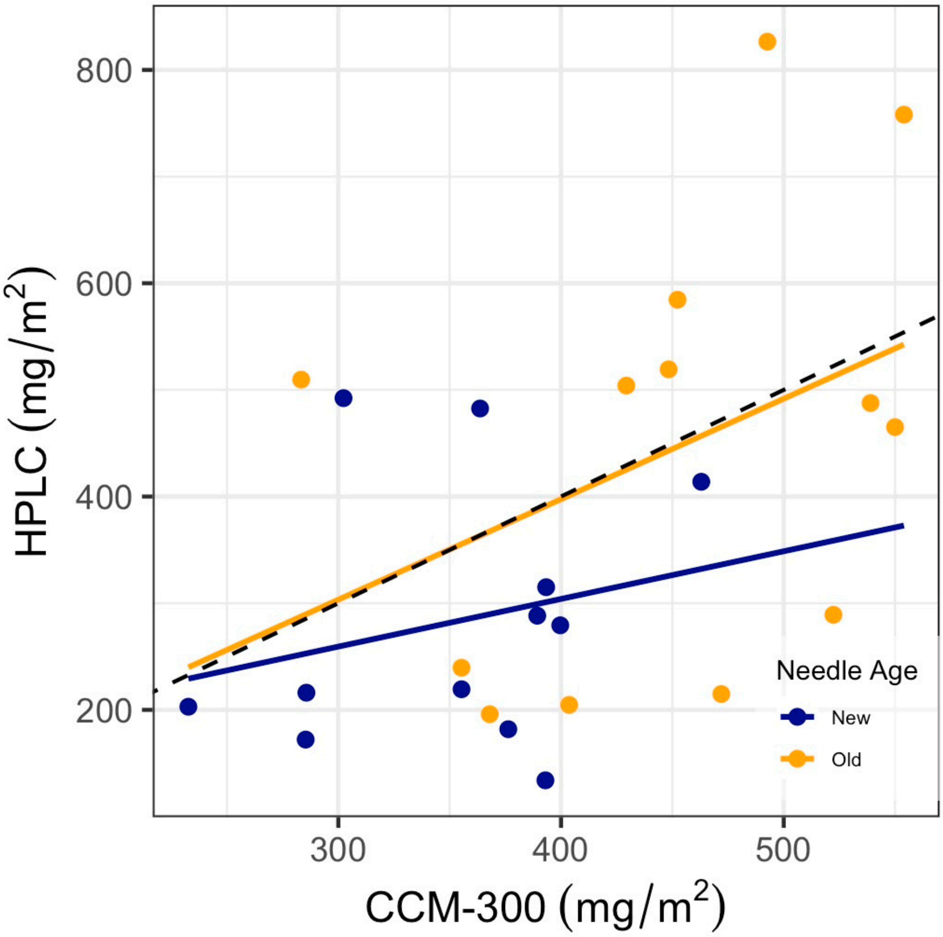

Figure S1. HPLC measurements from the UW-Madison dataset (n = 26).
